# Clinical Lectures on Diseases of the Eye

**Published:** 1864-04

**Authors:** E. L. Holmes

**Affiliations:** Chicago, Lecturer on Diseases of the Eye and Ear in Rush Medical College, and Surgeon of the Chicago Charitable Eye and Ear Infirmary


### CLINICAL LECTURES ON DISEASES OF THE EYE.

By E. L. HOLMES, M. D., of Chicago,
*Lecturer on Diseases of the Eye and Ear in Rush Medical College, and Surgeon of the Chicago Charitable Eye and Ear Infirmary.*

### THE CORNEA—INJURIES.

The diseases of the cornea are too numerous and too important to be dismissed with but few remarks, and yet three lectures must limit what I design to say upon the subject.

I shall ask your attention, to-day, to the injuries of the cornea, and in the two following lectures shall speak of acute and chronic inflammation of this membrane.

The cornea is liable to several kinds of injury, which you should well understand. One of the most common is that, produced by the presence of minute foreign bodies, lodged either on its surface or in its deeper layers. Millers and machinists are especially liable to this accident; often receiving particles of stone or steel upon the cornea. These minute bodies are often readily removed, in fact mechanics not unfrequently acquire considerable skill in removing them from the eyes of their fellow workmen. Simple as it may appear, it requires some degree of delicacy and steadiness of manipulation to remove these little bodies from the cornea. It even requires some practice to detect them, especially if they are light-colored. But with good eye-sight, and with the habit of examining the surface of the cornea, you can scarcely ever fail to recognize their presence. They can, perhaps, be seen most readily if you stand behind the patient, seated before a window, and look obliquely upon the cornea from above. I usually remove them with the patient in the same position. If the particles are simply lodged on the surface, or in the epithelium, I generally make use of a slender piece of hard wood, with quite a fine and yet slightly blunted point. With one hand the lids should be held apart, and with the other the point of the instrument should be carried gently against the particle, without scratching the cornea. If the foreign substance is deeply imbedded in the cornea, it is necessary to use some steel instrument, like a cataract needle with the point slightly dulled. It is surprising with what firmness the elastic tissue of the cornea grasps these little particles. Although the point of the instrument will readily lift some of them from their place, others can only be removed by repeated efforts—in fact they must be loosened on all sides—enucleated, as it were, before they can be dislodged. I have seen the most experienced oculists manipulate for more than a minute and not succeed in removing a piece of steel, imbedded in the cornea. Few patients possess sufficient fortitude to sit quietly through such an operation.

You will, therefore, generally remove particles deeply imbedded in the cornea, with less danger of injuring the eye, if you place the patient under the influence of chloroform. You then have perfect control of the eye.

Although I think it unnecessary to scrape the little wounds after removing pieces of iron or steel, for the purpose of clearing away the oxide that may remain, you should be aware that a steel instrument will sometime fracture a particle of cast iron or other hard substance. Care should be taken that every minute piece is taken away.

In very rare cases, where the particle is deep in the cornea, it may be advisable, first, to pass the point of a sharp lancet obliquely under the object. In this way the efforts to remove it with a cataract needle will be less liable to crowd the particle into the anterior chamber.

Occasionally you will meet with a case, where the fragment of metal or other hard substance has been thrown with such violence as to be forced through the cornea into the anterior chamber. The presence of such a substance in contact with the iris is very liable to produce troublesome iritis. It should be removed at once, by means of a fine pair of forceps, passed through an opening near the periphery of the cornea. If this cannot be accomplished without danger of bruising the iris, I think the piece of iris to which the foreign body is attached should be removed with the particle, as in the operation for artificial pupil, which I shall describe at some future time. I may here state, however, that the evulsion of a piece of the iris is less liable to cause iritis, than either the contusion of some of its fibres, or the presence of a minute foreign body upon its surface.

After the removal of bodies from the surface of the cornea little treatment is usually required. Rest, with the application of compresses, wet in cold water, is sufficient. It is surprising how rapidly these little wounds of the cornea generally heal. Sometimes, however, they do not heal readily, but become chronic ulcers. I shall hereafter allude to the treatment of such cases.

There is a class of accidents, which I have almost invariably found in the practice of others, and in my own experience to be exceedingly grave. I refer to severe burns, whether produced by boiling liquids, pieces of hot metal, caustic acids, or alkalies. The portion of the cornea in which the vitality is totally destroyed soon sloughs. The portions around this in a short time become the seat of a very severe inflammation which extends to the conjunctiva, and is accompanied with a muco-purulent discharge. Even where the burn is quite limited in extent, the cornea is often destroyed by the subsequent inflammation. One of the first symptoms of this inflammation is a loss of transparency, which becomes more and more marked, till a part or even the whole of the cornea becomes opaque. The cells thus modified are very apt to die, either slowly, as in ulceration, or rapidly, as in sloughing.

The prognosis in these cases, even when for a time apparently of little importance, should be cautiously given. The destructive inflammation may extend to other important parts of the eye, absolutely destroying vision, or the least that may be feared, it may be confined to the cornea, leaving an opacity encroaching more or less extensively over the pupil.

On being called to these cases, it should be your first care to remove all irritating substances from the eyes. This can be accomplished by a constant but gentle stream of cool water thrown between the lids. A solution of sugar and water is said to dissolve lime more readily than clear water. All foreign particles, like sand, ashes, or cinders, must be carefully removed. Few patients can bear the pain of thorough examination and cleansing of the eye in cases of burns, unless under the influence of chloroform. It is consequently always advisable to make use of this agent in the examination of those cases. Subsequent pain should be controlled by the use of anodynes internally, and cold compresses locally. Sometimes a couple of drops of a solution of neutral sulphate of atropine —grs. iv to the ounce—instilled between the lids every hour, will relieve pain. A few drops of pure castor oil, or olive oil, introduced in the same way, are soothing to the eyes, especially when particles of caustic remain under the lids. After the slough produced by the burn has been cast off, and inflammation well established, the muco-purulent discharge which attends it should be checked by the careful use of a solution of nitrate of silver, varying in strength from two to six grains to the ounce.

Punctured and incised wounds of the cornea, if so small as not to admit prolapsus of the iris, usually require little treatment, except rest for a few days, wet compresses and a spare diet. When the wound is near the centre of the cornea, and prolapsus of the iris has occurred, the use of atropine, as just directed, may draw the iris from the opening, especially if the incision is small. This plan will scarcely succeed if inflammation has already commenced. It is allowable immediately after an accident, if prolapsus of the iris exists, to endeavor to reduce it by gentle efforts with the end of a probe or other similar instrument. Although such efforts will occasionally be attended with wonderful results, it should be remembered that the iris seldom escapes injury by such a use of the probe. Traumatic iritis is very liable to follow such an injury. It is scarcely necesssary to say that the reduction of a prolapse of the iris should only be attempted after the patient is under the influence of chloroform. It is undoubtedly a better practice after a prolapsus has been established, to clip off the protruding portion of the iris with a delicate pair of scissors, especially if it projects far and is painful, and easily irritated by the motion of the lids. It might be well in certain cases to pass a fine ligature around the prolapsus, close to the cornea, as in changing the position of the pupil by irridesis. You are aware that the total destruction of small parts of the iris, either by removal with the knife or by ligature, is less dangerous to the iris and to the eye than even slight bruises. Hence the two little operations just described are usually less dangerous than efforts simply to replace the iris in the anterior chamber.

When the iris protrudes through a small opening near the periphery of the cornea, it might be well to try the effects of extract of Calabar bean, which possesses the property of producing strong contractions of the pupil. Large wounds parallel or nearly parallel with the periphery of the cornea, are less liable to produce prolapsus, and heal more readily than wounds of like extent nearer the centre. Such injuries almost always heal with loss of vision, although wonderful instances of perfect restoration of sight are reported in works on diseases of the eye. The edges should be adjusted as accurately as possible, and the same treatment pursued as after extraction of cataract. It is your duty to urge upon patients with even the slightest injuries of the cornea, the danger of exposure to too strong light, changes of temperature, or other causes of irritation. Injuries apparently the most insignificant have terminated in loss of sight. It is one of the most important points in the treatment of these cases, to direct particular attention to the state of the patient's health; in vitiated diathesis, to support and nourish rather than deplete. I believe mercury is almost never required in injuries of the cornea.

As works of reference upon this subject as well as upon injuries of the eye generally, I would refer you to the work of W. W. Cooper, on Injuries of the Eye, and to the chapters on injuries in the works of McKenzie, Lawrence, and Walton.

